# Main Ustilaginoidins and Their Distribution in Rice False Smut Balls

**DOI:** 10.3390/toxins7104023

**Published:** 2015-10-09

**Authors:** Jiajia Meng, Weibo Sun, Ziling Mao, Dan Xu, Xiaohan Wang, Shiqiong Lu, Daowan Lai, Yang Liu, Ligang Zhou, Guozhen Zhang

**Affiliations:** 1 College of Agronomy and Biotechnology, China Agricultural University, Beijing 100193, China; E-Mails: mengjiajiax@163.com (J.M.); sunweibo.1001@163.com (W.S.); maoziling2011@163.com (Z.M.); cauxudan@163.com (D.X.); wangxiaohan99@126.com (X.W.); shiqionglu@126.com (S.L.); dwlai@cau.edu.cn (D.L.); 2 Institute of Food Science and Technology, Chinese Academy of Agricultural Sciences/Key Laboratory of Agro-products Processing, Ministry of Agriculture, Beijing 100193, China; E-Mail: liuyang01@caas.cn

**Keywords:** ustilaginoidins, mycotoxin, phytotoxin, rice false smut balls, *Villosiclava virens*, *Ustilaginoidea virens*, HPLC

## Abstract

Rice false smut has become an increasingly serious fungal disease in rice (*Oryza sativa* L.) production worldwide. Ustilaginoidins are bis-naphtho-γ-pyrone mycotoxins previously isolated from the rice false smut balls (FSBs) infected by the pathogen *Villosiclava virens* in rice spikelets on panicles. To investigate the main ustilaginoidins and their distribution in rice FSBs, five main bis-naphtho-γ-pyrones, namely ustilaginoidins A (**1**), G (**2**), B (**3**), I (**4**) and C (**5**), were isolated and identified by NMR and high-resolution mass spectrometry as well as by comparison with the data in the literature. The rice FSBs at early, middle and late maturity stages were divided into their different parts and the contents of five main ustilaginoidins for each part were determined by HPLC analysis. The results revealed that the highest levels of ustilaginoidins were in late stage rice FSBs, followed by those at middle stage. Most ustilaginoidins, 96.4% of the total quantity, were distributed in the middle layer at early stage. However, ustilaginoidins were mainly distributed in the outer and middle layers at middle and late stages. Small amounts of ustilaginoidins A (**1**) and G (**2**) were found in the inner part of rice FSBs at each maturity stage. The contents of ustilaginoidins A (**1**) and G (**2**) without hydroxymethyl groups at C-2 and C-2’ of the γ-pyrone rings in rice FSBs were relatively high at early stage, while the contents of ustilaginoidins B (**3**), I (**4**), and C (**5**) with hydroxymethyl groups at C-2 or C-2’ were relatively high at late stage.

## 1. Introduction

Rice false smut, caused by *Villosiclava virens* (Nakata) Tanaka & Tanaka (anamorph: *Ustilaginoidea virens* Takahashi) [1], is one of the most destructive fungal diseases in many rice (*Oryza sativa* L.) cultivation areas over the past few years [2]. The infection of *V. virens* occurs in rice spikelets on panicles [3]. The fungus transforms grains into the ball-like colonies which we called false smut balls (FSBs). The color of rice FSBs gradually changes from white to yellow, then yellowish green, olive-green, and ultimately greenish-black during maturity [4,5]. Each mature FSB consists of dark-green chlamydospores (the outer layer), orange mycelia with immature chlamydospoes (the middle layer), white psudoparenchyma (the inner part) and glume. Rice false smut disease not only results in rice yield loss, but also contaminates rice grains and feed, and even more importantly, generates mycotoxins that are poisonous to humans and animals and creates concerns for food and feed safety [2,6].

It has been reported that rice FSBs and the false smut pathogen could produce two kinds of mycotoxins, namely ustiloxins and ustilaginoidins [7,8]. Ustiloxins are cyclic peptides which have been reported to have antimitotic activity by inhibiting microtubule assembly and cell skeleton formation of plant and animal cells [9,10,11,12,13,14,15]. Ustilaginoidins are bis-naphtho-γ-pyrone mycotoxins, and eighteen ustilaginoidins, namely isochaetochromin B_2_, ustilaginoidins A-P and E_1_, have been isolated so far [7,8,16]. They exhibit a variety of biological activities such as cytotoxic activity [17,18,19,20], antibacterial activity [8,21], inhibitory activity on HIV-1 integrase [22], phytotoxic activity [8,23,24], and inhibitory activity on triacylglycerol synthesis in mammalian cells [25].

The main ustiloxins (*i.e*., ustiloxins A and B) can be purified from rice FSBs by macroporous resins in combination with a hydrophilic C_18_ (ODS-AQ) column chromatography [26,27]. Moreover, ustiloxins can be detected by the methods of high-performance liquid chromatography (HPLC) [27,28], liquid chromatography-mass spectrometry (LC-MS) [27], and enzyme-linked immunosorbent assay (ELISA) [29,30]. To our knowledge, there are no reports on the identification of the main ustilaginoidins and their distribution in rice FSBs or on methods for detecting the contents of ustilaginoidins in samples. In this study, five main ustilaginoidins were isolated and identified from rice FSBs. The contents of the main ustilaginoidins in the samples were analyzed by HPLC. The distribution of ustilaginoidins as well as their contents in different parts (*i.e*., glume, outer layer, middle layer and inner part) of rice FSBs were also clarified. The results will provide the basis for isolation and analysis of the ustilagiloidins in rice samples, as well as for revealing metabolic pathways, physiological and ecological functions of the ustilaginoidins in rice FSBs.

## 2. Results and Discussion

### 2.1. Characterization and Analysis of the Main Ustilaginoidins in Rice FSBs

**Figure 1:**
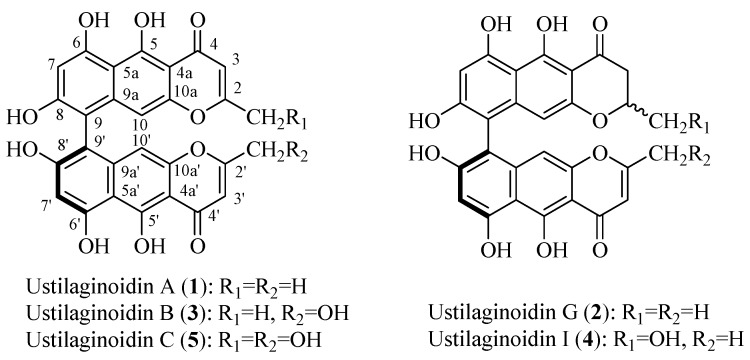
Chemical structures of the five main ustilaginoidins.

After repeated column chromatographic purification using Sephadex LH-20, preparative high-speed counter-current chromatography (HSCCC), and semi-preparative HPLC, the ethyl acetate extract of rice FSBs afforded five main compounds **1**–**5**. After comparing their physicochemical and spectrometric data with those reported in the literature [8,16], they were identified as ustilaginoidins A (**1**), G (**2**), B (**3**), I (**4**) and C (**5**), whose structures are shown in Figure 1 and their UV absorption spectra are shown in Supplementary Figure S1.

**Figure 2:**
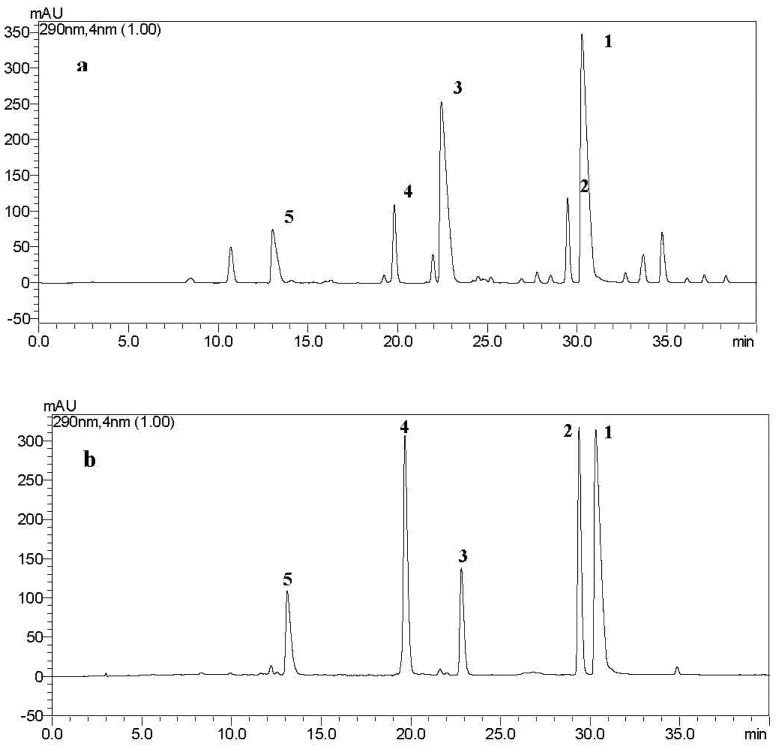
HPLC analysis of ustilaginoidins A (**1**), G (**2**), B (**3**), I (**4**) and C (**5**) in rice FSBs. (**a**) is the HPLC profile of the ethyl acetate extract from the mycelia. Arabic numerals **1**–**5** in the figure represent ustilaginoidins A, G, B, I and C, respectively; (**b**) is the HPLC profile of the standard ustilaginoidins A, G, B, I and C. The retention times of ustilaginoidins C, I, B, G and A were 13.10, 19.67, 22.82, 29.39 and 30.32 min, respectively.

Figure 2 shows the HPLC profiles of the ethyl acetate extract and standard ustilaginoidins A (**1**), G (**2**), B (**3**), I (**4**) and C (**5**), respectively. The main ustilaginoidins A, G, B, I and C in rice FSBs were identified by comparison of their retention times with the standard ustilaginoidins as well as the UV absorption spectra. HPLC analysis was completed in 40 min.

Based on the above results, the five main ustilaginoidins were selected for quantitative analysis by HPLC. The linear equations for ustilaginoidins A, G, B, I and C are shown in Table 1. The results showed that the developed HPLC method had good linearity within the range of 0.03125–1.5 μg in the sample injected each time.

**Table 1:** The linear equations of five main ustilaginoidins by HPLC analysis.

Ustilaginoidin	Retention Time (min)	Linear equation *Y* = a*X* + b	Correlation coefficient (*R*^2^)
Ustilaginoidin A (**1**)	30.32	*Y* = 6156397.5754 *X* − 33957.4836	0.9995
Ustilaginoidin G (**2**)	29.39	*Y* = 6279322.9760 *X* − 7659.1240	0.9985
Ustilaginoidin B (**3**)	22.82	*Y* = 7087462.4058 *X* + 263979.1177	0.9996
Ustilaginlodin I (**4**)	19.67	*Y* = 4858545.7034 *X* + 128004.6527	0.9986
Ustilaginoidin C (**5**)	13.10	*Y* = 6189302.6749 *X* + 35291.4332	0.9993

*Y* is the peak area, *X* is the quantity (μg) of the sample injected each time, and *R*^2^ is the correlation coefficient.

### 2.2. Distribution of Main Ustilaginoidins in Rice FSBs

Table 2 shows the contents of the five main ustilaginoidins in different parts of rice FSBs at early, middle and late maturity stages. We considered the sum of the five main ustilaginoidins (*i.e*., ustilaginoidins A, G, B, I and C) as the total content of ustilaginoidins in each sample though there were some other minor ustilaginoidins detected in the crude extract (Figure 2A). With the increasing of maturity degree, the FSBs enlarged gradually. The total average dry weight for each FSB was 32.9, 97.7 and 151.3 mg, respectively. Meanwhile, the total ustilaginoidin quantity for each FSB increased with maturity degree as well, from 0.6 to 5.5 and finally to 11.8 mg, respectively. Moreover, we found that the ustilaginoidins were distributed mainly in the middle layer at early stage, accounting for 96.4% of the total ustilaginoidin quantity, whereas at the mid and late stages, the ustilaginoidins exhibited a distribution mostly in the outer and middle layers (Table 2).

The levels of ustilaginoidins in the inner part of the FSBs were significantly lower than those in the outer and middle layer at all stages (Table 2). However, the levels of ustilaginoidins in the inner parts increased with the maturity increase of rice FSBs. The contents were 0.9, 1.0 and 1.8 mg/g at early, middle and late stage, respectively.

When the chlamydospores emerged, the contents of ustilaginoidins B (**3**), I (**4**) and C (**5**) in the outer layer (33.9, 10.1 and 12.1 mg/g at middle stage, and 36.8, 15.1 and 12.5 mg/g at late stage, respectively) were significantly higher than those in the middle layer (21.7, 5.9 and 5.4 mg/g at middle stage, and 29.8, 8.7 and 9.0 mg/g at late stage, respectively), and contents of these three ustilaginoidins reached the maximum values at late stage. However, the contents of ustilaginoidins A (**1**) and G (**2**) at middle stage were almost equal to those at late stage. The total content of ustilaginoidins in the outer layer was higher than that in the middle layer.

The present study showed that total ustilaginoidin content maximized at late maturity stage (Table 2), and it increased significantly at each increased maturity degree, indicating that levels of ustilaginoidins depend on the maturity stage of rice grains. In addition, we found that though the content of each ustilaginoidin in inner part was very low, it increased gradually with maturity increases. Interestingly, the contents of ustilaginoidins B (**3**), I (**4**), and C (**5**) with relatively big polarity were low at early stage, and their contents increased significantly at late stage. By contrast, the contents of ustilaginoidins A (**1**) and G (**2**) with relatively small polarity were high at early stage, and their contents decreased at late stage (Table 2). This indicates that ustilaginoidin A (**1**) was oxidized at C-2 or C-2’ of the γ-pyrone rings into ustilaginoidins B (**3**) and C (**5**) containing one or two hydroxymethyl groups, and ustilaginoidin G (**2**) was oxidized at C-2’ into ustilaginoidin I (**4**), which suggests that both ustilaginoidins A (**1**) and G (**2**) should be the precursors of ustilaginoidins B (**3**), I (**4**), and C (**5**), a prediction that needs to be verified. By comparing the ustilaginoidins isolated from rice FSBs and pathogen fermentation cultures, 13 ustilaginoidins (*i.e*., ustilaginoidins A, D, E, F, G, E_1_, K-P, and isochaetochromin B_2_) isolated from the fermentation cultures of rice false smut pathogen were shown to be low-oxidative-degree ustilaginoidins without hydroxymethyl groups at C-2 or C-2’ [8]. Ustilaginoidins B, C, H, I and J, which had a high degree of oxidation with hydroxymethyl groups at C-2 or C-2’ of the γ-pyrone rings, were only found in rice FSBs [16]. In this study, ustilaginoidins B, I and C were found in high concentrations in the outer layer of the FSBs, indicating that these high-oxidative-degree ustilaginoidins may have important biological functions that need to be investigated in detail. The genomic data of *V. virens* indicated that some transcriptional genes involved in biosynthesis of the secondary metabolites including ustilaginoidins were highly enhanced during early infection and thus were speculated to play vital roles [31]. However, the interaction between *V. virens* and its host as well as the conversion process of the ustilaginoidins were still unclear.

**Table 2:** Contents of main ustilaginoidins in rice FSBs at different maturity stages.

Part of FSB	Average weight in each FSB (mg)	Ustilginoidin content (mg/g)					
		Ustilginoidin A (1)	Ustilginoidin G (2)	Ustilginoidin B (3)	Ustilginoidin I (4)	Ustilginoidin C (5)	Total
**Early stage**							
Outer layer	Nd	nd	nd	nd	nd	nd	nd
Middle layer	5.7 ± 0.4 ef	60.4 ± 5.8 a	5.4 ± 0.1 c	21.9 ± 2.6 c	3.9 ± 0.3 c	4.1 ± 0.5 d	95.7 ± 9.2 b
Inner part	22.9 ± 1.3 d	0.7 ± 0.1 d	0.2 ± 0.0 d	nd	nd	nd	0.9 ± 0.1 c
Glume	4.3 ± 0.6 f	nd	nd	nd	nd	nd	nd
**Middle stage**							
Outer layer	14.1 ± 3.0 de	54.7 ± 1.8 ab	6.3 ± 0.1 b	33.9 ± 1.2 ab	10.1 ± 0.5 b	12.1 ± 0.8 a	117.1 ± 3.9 a
Middle layer	38.7 ± 7.5 c	56.0 ± 5.9 ab	8.7 ± 0.3 a	21.7 ± 2.2 c	5.9 ± 0.7 c	5.4 ± 0.5 c	97.4 ± 8.8 b
Inner part	41.6 ± 5.8 bc	0.6 ± 0.1 d	0.4 ± 0.1 d	nd	nd	nd	1.0 ± 0.2 c
Glume	3.3 ± 0.6 f	nd	nd	nd	nd	nd	nd
**Late stage**							
Outer layer	49.3 ± 3.0 ab	44.9 ± 5.5 c	6.0 ± 1.0 bc	36.8 ± 3.9 a	15.1 ± 1.9 a	12.5 ± 1.2 a	115.4 ± 13.5 a
Middle layer	54.7 ± 12.8 a	54.0 ± 2.1 b	9.3 ± 0.3 a	29.8 ± 2.5 b	8.7 ± 2.3 b	9.0 ± 0.2 b	110.8 ± 6.6 a
Inner part	44.1 ± 6.3 bc	1.2 ± 0.3 d	0.6 ± 0.1 d	nd	nd	nd	1.8 ± 0.4 c
Glume	3.2 ± 0.2 f	nd	nd	nd	nd	nd	nd

FSB: false smut ball; Each value represents the mean of triplicate ± standard deviations. Different letters indicate significant differences among different maturity sates in each column at *p* ≤ 0.05. nd: not detected.

## 3. Experimental Section

### 3.1. General

HR-ESI-MS spectra were recorded on a Bruker Apex IV FTMS instrument (Bruker Daltonics, Bremen, Germany). ^1^H and ^13^C-NMR spectra were measured on Bruker Avance 600 NMR spectrometers (^1^H at 600 MHz and ^13^C at 150 MHz) (Bruker BioSpin, Zurich, Switzerland). Chemical shifts were expressed in δ (ppm) relative to tetramethylsilane (TMS) as an internal standard. Preparative high-speed counter-current chromatography (HSCCC) was performed on a TBE-300B instrument (Tauto Biotech, Shanghai, China), equipped with three preparative coils, a polytetrafluoroethylene tube (2.6 mm in diameter, and total volume of 300 mL), and a 20-mL sample loop. The separation was carried out at 25 °C using a two-phase solvent system, at a flow rate of 3.2 mL/min, revolution speed of 800 rpm, and detection wavelength at 280 nm. Semi-preparative HPLC separation was carried out on a Lumtech instrument (Lumiere Tech. Ltd., Beijing, China) equipped with a K-501 pump (flow rate was 3 mL/min) and a K-2501 UV detector (detection was set at 290 nm), using a Luna-C_18_ column (250 mm × 10 mm i.d., 5 μm, Phenomenex Inc., Torrance, CA, USA). A Shimadzu Prominence LC-20A high-performance liquid chromatography system (Kyoto, Japan) was consisted of two LC-20AT solvent delivery units, an SIL-20A autosampler, an SPD-M20A photodiode array detector, a CBM-20Alite system controller, and a reversed-phase Luna C_18_ column (250 mm × 4.6 mm, 5 μm) (Phenomenex, Torrance, CA, USA). An electric heating constant temperature incubator was purchased from Tianjin Zhonghuan Experiment Electric Stove Co. Ltd. (Tianjin, China). An ultrasonic cleaner (KH-500E, Kunshan, China) was purchased from Kunshan Hechuang Ultrasonic Apparatus Co. Ltd.

Silica gel (200-300 mesh) for column chromatography was purchased from the Qingdao Marine Chemical Company (Qingdao, China). Sephadex LH-20 was purchased from Pharmacia Biotech, Sweden. All other chemicals and reagents were of analytical grade.

### 3.2. Rice False Smut Balls

The rice false smut balls (FSBs) were collected from Chengdu (104.1°E, 30.7°N) in the Sichuan Province of China in 2014. The materials were left to dry in shade at room temperature to a constant weight, and were then stored in sealed plastic bags at −20 °C until required.

**Figure 3:**
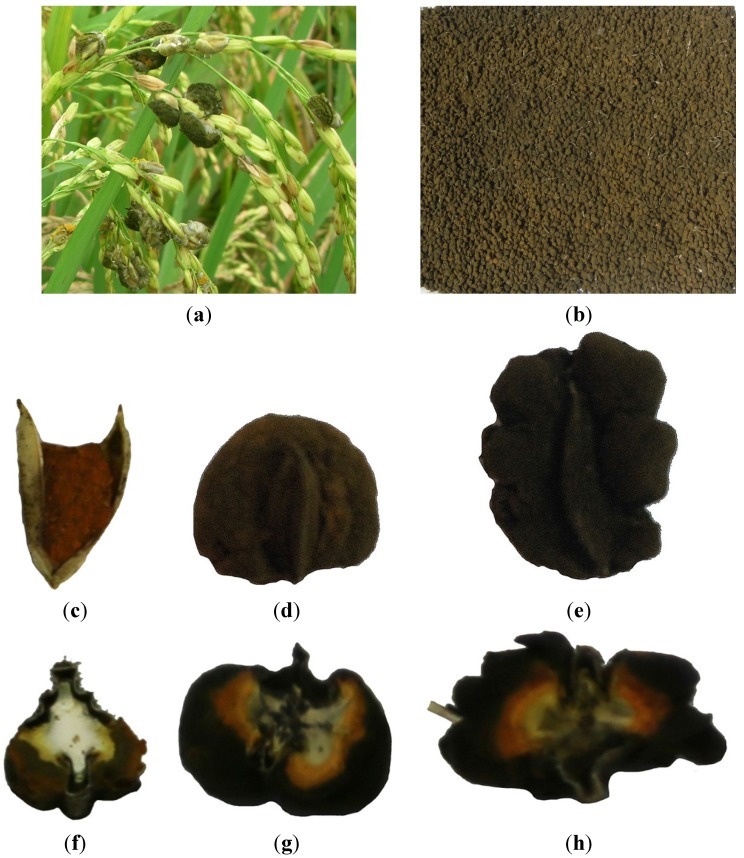
Rice false smut balls (FSBs) and their sections. (**a**) Matured FSBs in rice plants; (**b**) Rice FSBs collected from rice plants infected with false smut disease; (**c**) Whole FSB at early stage; (**d**) Whole FSB at middle stage; (**e**) Whole FSB at late stage; (**f**) Section of rice FSB at early stage. (**g**) Section of rice FSB at middle stage. (**h**) Section of rice FSB at late stage.

The color of the rice FSBs at early stage was white to yellow, and the average weight for each ball was 32.9 mg. At middle stage, the average weight for each ball was about 97.7 mg, and the balls became yellowish green covered by a powder of dark-green chlamydospores. Lastly, the color of rice FSBs was greenish black, and the balls became much bigger (average weight for each ball was 151.3 mg) with cracks on the surface. The matured FSBs in rice plants as well as the whole balls at the early, middle and late stages, and their corresponding sections are shown in Figure 3.

The rice FSBs at different maturity stages were carefully divided into the parts of chlamydospores (outer layer), mycelia with immature chlamydospores (middle layer), psudoparenchyma (inner part) and glume by tweezer and scalpel. All samples were weighed and ground, and then kept at 4 °C until analysis.

### 3.3. Extraction, Fractionation and Identification of the Ustilaginoidins

The dry and powdered FSBs (9.1 kg) were soaked in deionized water at room temperature three times (3 × 30 L, 48 h for each time). After filtration, the residue was soaked in ethanol at room temperature another three times (3 × 30 L, 48 h for each time). The ethanol filtrates were combined and concentrated in a vacuum to obtain a black gum substance which was suspended in water and extracted first with the equal volume of petroleum ether, then with ethyl acetate (EtOAc), and last with *n*-butanol for three repetitions. The combined EtOAc solution was concentrated to obtain the crude EtOAc extract (264.09 g) which was first chromatographed on a silica gel column eluted with a step gradient of CH_2_Cl_2_-EtOAc (1:0, 100:1, 10:1, 1:1, and 0:1, *v/v*) to yield two main fractions Fr.A (27.5 g) and Fr.B (43.5 g). Fr.A (27.5 g) was performed on high-speed counter-current chromatography (HSCCC) with a two-phase solvent system composed of *n*-hexane-ethyl acetate-methanol-water (6.5:3.5:5.0:5.0, *v/v*) to yield two peak fractions which were further purified by semi-preparative HPLC under a methanol:water volume ratio of 75:25 to obtain ustilaginoidins A (**1**, 11.5 mg) and G (**2**, 9.3 mg). Fr.B (10.0 g) was fractionated on a Sephadex LH-20 column eluted with CHCl_3_-CH_3_OH (1:1, *v/v*) to afford Fr.B1 (5.2 g) and Fr.B2 (3.6 g). Fr.B1 was performed on HSCCC with two-phase solvent system composed of *n*-hexane-ethyl acetate-methanol-water (4.0:5.0:5.0:6.0, *v/v*) to yield two peak fractions which were further purified by semi-preparative HPLC under a methanol:water volume ratio of 65:35 to obtain ustilaginoidins B (**3**, 10.0 mg) and I (**4**, 10.8 mg). Fr.B2 was performed on HSCCC with two-phase solvent system composed of *n*-hexane-ethyl acetate-methanol-water (3.0:5.0:4.0:6.7, *v/v*) to yield one peak fraction which was further purified by semi-preparative HPLC under a methanol:water volume ratio of 50:50 to obtain ustilaginoidin C (**5**, 4.6 mg).

All the purified compounds were isolated as red or yellow amorphous powder. The molecular formula C_28_H_18_O_10_ of ustilaginoidin A (**1**) was assigned by HR-ESI-MS, *m*/*z* 513.0831 [M − H]^−^ (calculated for C_28_H_17_O_10_, 513.0827). Similarly, molecular formula C_28_H_20_O_10_ of ustilaginoidin G (**2**) was assigned by HR-ESI-MS, *m*/z 515.0987 [M − H]^−^ (calculated for C_28_H_19_O_10_, 515.0984); molecular formula C_28_H_18_O_11_ of ustilaginoidin B (**3**) was assigned by HR-ESI-MS, *m*/*z* 531.0903 [M + H]^+^ (calculated for C_28_H_19_O_11_, 531.0922); molecular formula C_28_H_20_O_11_ of ustilaginoidin I (**4**) was assigned by HR-ESI-MS, *m*/*z* 533.1070 [M + H]^+^ (calculated for 533.1078); molecular formula C_28_H_18_O_12_ of ustilaginoidin C (**5**) was assigned by HR-ESI-MS, *m*/*z* 547.0856 [M + H]^+^ (calculated for C_28_H_19_O_12_, 547.0871). The ^1^H NMR (600 MHz) and ^13^C NMR (150 MHz) data of the compounds were shown in Supplementary Tables S1 and S2 of Supplementary Materials. All the data were consistent with those in literature [8,16].

### 3.4. HPLC Analysis of Main Ustilaginoidins in Rice FSBs

The powdery sample (10 mg) was weighed and then extracted with ethyl acetate for three times (3 × 1 mL, 20 min for each time) in an ultrasonic bath at room temperature. The ethyl acetate extract was concentrated by a rotary evaporator to dryness under vacuum at 28 °C. The obtained residue was dissolved in 3 mL of methanol. It was then filtered through a microporous filter (pore size, 0.22 μm) before analysis.

1 mg of the purified ustilaginoidin was dissolved in 1 mL of methanol to obtain the mother solution (1 mg/mL) which was further diluted into a series of concentrations of 150, 100, 50, 31.25, 25, 12.5, 6.25 and 3.125 μg/mL with methanol, and the diluted solutions were kept at 4 °C. Each solution was filtered and analyzed by an HPLC system eluted with a linear gradient of methanol from 50 to 100% (*v/v*) and water (containing 0.01% oxalic acid) from 50 to 0% (*v/v*) over 40 min at a flow rate of 1.0 mL/min. The temperature was maintained at 30 °C, UV detection at 290 nm, and the sample injection volume at 10 μL. The LC-solution multi-PDA workstation was employed to acquire and process chromatographic data.

### 3.5. Statistical Analysis

All tests were performed with three replications, and the results were represented by their mean values and the standard deviations (SD). Statistical analysis of the data was carried out using analysis of variance (one-way ANOVA) to detect significant differences by PROC ANOVA of SAS version 8.2 (SAS Institute, Cary, NC, USA). The term significant was used to denote the differences at *p* ≤ 0.05.

## 4. Conclusions

In this study, ustilaginoidins A (**1**), G (**2**), B (**3**), I (**4**) and C (**5**) as the main bis-naphtho-γ-pyrone mycotoxins were isolated and identified in the ethyl acetate extract from rice false smut balls (FSBs). The contents of the ustilaginoidins in rice FSBs increased with the increase of maturity degree. At early maturity stage, ustilaginoidins were mainly distributed in the middle layer with a proportion of 96.4%, while at middle and late stages, ustilaginoidins were mainly distributed in the middle and outer layer. Very low levels of ustilaginoidins were detected in the inner layer of rice FSBs at each maturity stage. The contents of ustilaginoidins A and G in rice FSBs, with a low degree of oxidation, were relatively high at early stage, while the contents of ustilaginoidins B, I, and C, with a high degree of oxidation, were relatively high at late stage. This indicated that both ustilaginoidins A and G should be the precursors of ustilaginoidins B, I, and C, something which needs to be verified. Isolation and structural identification of other ustilaginoidins from rice FSBs are in progress. The physiological and ecological functions of ustilaginoidins in rice FSBs need to be studied in detail.

## Supplementary Materials

Supplementary materials can be accessed at: http://www.mdpi.com/2072-6651/7/10/4023/s1.
