# Supplementary material for: Main Ustilaginoidins and Their Distribution in Rice False Smut Balls
Source: Toxins (Basel). 2015 Oct 9;7(10):4023–34. doi: 10.3390/toxins7104023 (PMC4626718; doi:10.3390/toxins7104023)
Supplement: Supplementary file 1 [file toxins-07-04023-s001.pdf]

Supplementary Information

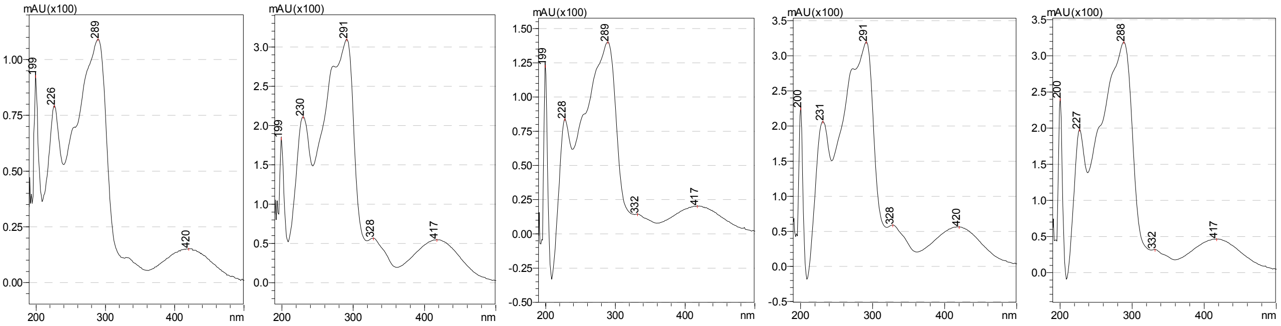

(**a**) (**b**) (**c**) (**d**) (**e**)

Figure S1. UV absorption spectra of ustilaginoidins C (**a**), I (**b**), B (**c**), G (**d**) and A (**e**).

Table S1. The data of ^{13}C NMR and ^{1}H NMR for ustilaginoidins A (**1**) and G (**2**).

**Position**	**Ustilaginoidin A (1)**		**Ustilaginoidin G (2)**	
	**^{13}C NMR (150 MHz)**	**^{1}H NMR (600 MHz)**	**^{13}C NMR (150 MHz)**	**^{1}H NMR (600 MHz)**
2, 2’	171.0	-	74.1, 171.0	4.42 ddq (11.9, 2.4, 5.9)
3, 3’	106.3	6.11 s	43.6, 105.3	2.80 dd (17.4, 11.9)<br>2.72 dd (17.2, 2.4)<br>5.90 s
4, 4’	184.7	-	184.7, 199.4	-
4a, 4a’	102.8	-	102.7, 102.7	-
5, 5’	160.7	-	161.7, 163.2	-
5a, 5a’	106.6	-	102.8, 105.3	-
6, 6’	154.6	-	156.6, 159.6	-
7, 7’	99.6	6.62 s	99.6, 100.1	6.50 s<br>6.60 s
8, 8’	159.7	-	160.6, 161.6	-
9, 9’	107.0	-	106.6, 106.9	-
9a, 9a’	141.2	-	141.1, 143.1	-
10, 10’	101.5	6.36 s	100.7, 101.4	6.11 s<br>6.38 s
10a, 10a’	153.4	-	153.4, 154.6	-
2, 2’-CH_3	20.6	2.30 s	20.6, 20.9	1.34 d (5.9)<br>2.31 s

Note: Ustilaginoidins A (**1**) and G (**2**) were measured in acetone-d_6, Chemical shifts were given on the δ (ppm) scale with TMS as the internal standard and coupling constants (J) were given in Hz. The letters s, d, dd and ddq meant singlet, doublet, doublet of doublets, and doublet of doublet of quartets, respectively in NMR spectrum. The letters a and a’ meant the positions in the chemical structures shown in Figure 1.

Table S2. The data of ^{13}C NMR and ^{1}H NMR for ustilaginoidins B (**3**), I (**4**) and C (**5**).

Position	Ustilaginoidin B (3)		Ustilaginoidin I (4)		Ustilaginoidin C (5)	
	^{13}C NMR (150 MHz)	^{1}H NMR (600 MHz)	^{13}C NMR (150 MHz)	^{1}H NMR (600 MHz)	^{13}C NMR (150 MHz)	^{1}H NMR (600 MHz)
2, 2’	169.7<br>172.4	-	77.3<br>169.8	4.34 d (9.5)	172.4	-
3, 3’	103.4<br>105.9	6.16 s<br>6.16 s	37.7<br>105.8	2.92 dd (17.3, 12.5)<br>2.61 d (17.2)<br>5.70 s	103.3	6.17 s
4, 4’	183.3<br>183.4	-	183.3<br>198.0	-	183.4	-
4a, 4a’	101.7<br>102.2	-	101.7<br>101.8	-	102.1	-
5, 5’	162.7<br>162.7	-	162.6<br>164.7	-	162.6	-
5a, 5a’	105.9<br>106.4	-	104.3<br>105.8	-	105.9	-
6, 6’	158.1<br>158.1	-	157.9<br>158.8	-	158.0	-
7, 7’	98.0<br>98.2	6.59 s<br>6.60 s	98.0<br>98.5	6.47 s<br>6.57 s	98.2	6.61 s
8, 8’	159.1<br>159.2	-	159.0<br>160.1	-	159.2	-
9, 9’	106.4<br>106.4	-	106.6<br>106.9	-	106.4	-
9a, 9a’	139.8<br>140.0	-	139.7<br>141.6	-	139.9	-
10, 10’	100.8<br>100.8	6.16 s<br>6.20 s	100.0<br>100.7	6.17 s<br>6.20 s	100.9	6.20 s
10a, 10a’	151.7<br>152.0	-	151.9<br>154.8	-	151.6	-
2, 2’-CH_3	20.2	2.24 s	20.2	2.27 s	-	-
2, 2’-C$\underline{H}_2$OH	-	4.27 s	-	3.53 dd (18.4, 2.8)	-	4.27 d (4.4)
2, 2’-CH_2O$\underline{H}$	-	5.76 s	-	4.98 br.s	-	nd
6, 6’-OH	-	10.03 s<br>10.03 s	-	9.97 s<br>2H	-	9.97 s
8, 8’-OH	-	9.82 s<br>9.82 s	-	9.79 s<br>9.78 s	-	9.82 s
2, 2’-$\underline{C}H_2$OH	59.8	-	62.7	-	59.8	-

Note: Ustilaginoidins B (**3**), I (**4**) and C (**5**) were measured in DMSO-d_6. Chemical shifts were given on the δ (ppm) scale with TMS as the internal standard and coupling constants (J) were given in Hz. nd: not detected. The letters s, d, dd and br.s meant singlet, doublet, doublet of doublets, and broad-singlet, respectively in NMR spectrum. The letters a and a’ meant the positions in the chemical structures shown in Figure 1.
